# Prevention of hazardous use of alcohol among high school students: a study protocol for the randomized controlled trial ‘Our choice’

**DOI:** 10.1186/s12889-023-16976-y

**Published:** 2023-10-24

**Authors:** Kristine Rømer Thomsen, Lotte Vallentin-Holbech, Synnøve Xylander, Kaare Bro Wellnitz, Janne Tolstrup, Anette Søgaard Nielsen, Sarah W. Feldstein Ewing

**Affiliations:** 1 Centre for Alcohol and Drug Research, Aarhus BSS, Aarhus University, Bartholins Allé 10, building 1322, Aarhus, DK-8000 Denmark; 2 Research Clinic for Functional Disorders and Psychosomatics, Aarhus University Hospital, Aarhus, Denmark; 3 Department of Clinical Medicine, Aarhus University, Aarhus, Denmark; 4 National Institute of Public Health, University of Southern Denmark, Copenhagen, Denmark; 5 Unit for Clinical Alcohol Research, Clinical Institute, Psychiatric University Hospital, University of Southern Denmark, Odense, Denmark; 6 Department of Psychology, University of Rhode Island, Kingston, USA

**Keywords:** Prevention, Alcohol, Substances, Adolescents, Motivational interviewing, Parents, School policies, High school

## Abstract

**Background:**

Adolescent hazardous alcohol use is prevalent and has serious short- and long-term consequences. The trial ‘Our Choice’ examines efficacy, feasibility and acceptability of prevention interventions targeting school, parent, and student levels at Danish high schools. We hypothesize that students in a structural intervention (school and parent levels) reduce hazardous alcohol use and related health behaviors compared to students in an assessment only control group 12 months post baseline; and that adding group-based Motivational Interviewing (group MI) yields further improvements. The study examines the efficacy of interventions targeting multiple levels with the aim of providing novel insights into prevention of adolescent hazardous alcohol use and related health outcomes.

**Method:**

The study employs a parallel group cluster randomized controlled trial design with three conditions: (1) structural condition targeting school and parent levels, (2) structural condition combined with group MI which also targets the student level, and (3) assessment-only control condition. A participatory approach is used to adapt and develop interventions. Sixteen high schools in Denmark and about N = 3100 first-year students (15–18 years) enrolled in high school in August 2023 will be recruited. Data will be collected via online questionnaires pre-interventions (baseline), 2, 6, 9 and 12 month post baseline and analyzed with generalized linear mixed models. The primary outcome is past month high intensity drinking; secondary outcomes are alcohol use, alcohol-related consequences, well-being, tobacco, and illegal substance use. Feasibility and acceptability will be assessed via surveys (students) and interviews (high school staff) to inform future implementation.

**Discussion:**

‘Our Choice’ is the first trial to compare the efficacy of a structural intervention targeting school- and parent levels to an intervention targeting these levels and the student level via group MI – on hazardous drinking and related health outcomes among students. Preventing and reducing hazardous alcohol use during adolescence is crucial due to the short- and long-term negative consequences. The tested interventions can be implemented at low cost. The study has significant implications for adolescent health and well-being and has potential to inform evidence-based decisions on alcohol prevention policy, education, and health professions.

**Trial Registration Number:**

The trial was retrospectively registered at ClinicalTrials.gov on August 24th, 2023. Trial Registration Number: ID NCT06018389.

**Supplementary Information:**

The online version contains supplementary material available at 10.1186/s12889-023-16976-y.

## Background

### Heavy drinking is widespread and an integral part of high school students’ social life

Alcohol continues to be the top substance used by adolescents across the globe [1, 2]. Danish adolescents are no exception; they have some of the highest rates of alcohol consumption worldwide: among 15-16-year Danes 40% report past month intoxication, which is the highest rate of all European countries [3].

Among Danish youth who drink, many experience negative consequences related to their drinking. In a recent survey [4], 16–17 year old Danes reported high levels of past year negative consequences related to drinking, including alcohol-related accidents and injuries such as bike crashes (26.3%); intimate and sexual experiences, which they later regret (kissing 31.1%; sex 9%); sending texts or sharing something on social media, which they later regret (25.7%); taking illegal drugs, which they later regret (3.7%); and missing out school or work (12.9%).

In Danish high schools (in Danish: Almen studentereksamen, STX) drinking is an integral part of the students’ social life, and to a larger degree than in other types of publicly funded schooling (e.g. vocational education [5]). Several reports document how young Danes increase, or initiate alcohol use shortly after admission to high school [6–8]. For example, 64% of Danish youngsters reported that they increased or initiated their alcohol use during the transition to high school [8].

Despite high rates of heavy drinking in Danish adolescents [9, 10], most adolescents in Denmark (and their parents [11]) rarely perceive adolescent alcohol use as a problem or something that needs to be reduced [6, 12, 13]. At the same time, very few adolescents seek or receive help for their hazardous alcohol use [14–17]. This is illustrated by adolescents representing less than 1% of all admissions to publicly funded treatment for alcohol problems in Denmark [17]. Furthermore, prevention programs are typically not an integral part of middle and high schools in Denmark. In short, we lack knowledge on which substance use prevention interventions are effective in Danish school settings.

### Use of other substances than alcohol

In the past decade prevalence of cigarette use in Denmark has declined, also among adolescents [18], which has been attributed to public health initiatives, increased awareness of risks related to smoking, and stricter tobacco regulations [19, 20]. Following this progress, nicotine-containing products like e-cigarettes, vaping devices, and smokeless options (e.g., snus, chewing tobacco, pouches) have surged in popularity as tobacco alternatives. These alternatives raise concerns due to the appeal of flavors masking nicotine’s harshness, and the potential risk that these alternatives may act as gateway to traditional smoking or lead to dual use [21].

Danish adolescents have the highest cannabis use among all of the Nordic countries [3, 22]. Among 15-25-year-olds: 20% report past year use and 9% past month use [23]. Concerns about adolescent cannabis use have been magnified due to the dramatic increases in Δ-9-tetrahydrocannabinol (THC; the main psychoactive component) concentration in cannabis products across Europe and the U.S [24, 25]. We found an alarming 3-fold increase in THC concentration in Danish cannabis resin from 8% in year 2000 to 25% in 2017 [26], the highest concentration throughout Europe [25]. This is concerning, as high THC levels have detrimental effects on cognitive function and mental health [27–29], including cannabis-induced psychosis [30], and is linked with increases in admission to cannabis use disorder treatment in Europe [31] and in Denmark [32]. Of note, the most popular types of cannabis in Denmark are cannabis resin and skunk [23], which have high levels of THC [33].

The second most commonly used illegal drug in Denmark is cocaine [34]. About 3% of young adults in Denmark report past year use of cocaine, the third highest prevalence among European countries in the EMCDDA [35]. Concerns about adolescent cocaine use have been magnified recently due to reported increases in purity across Europe [35] and Denmark, where we found a marked increase since 2016 [36].

Although use of cannabis is more prevalent in vocational school settings than in high school settings in Denmark [5], the prevalence of use of cannabis and other illegal substances increases from middle school to high school. In our previous study of 515 high school students, 14% had used cannabis and 3% reported past month cannabis use. Furthermore, heavy use of alcohol has been shown to increase risk of illegal substance use [37].

### Prevention interventions in high schools

Current evidence suggests that a socio-ecological approach that emphasizes the structural context while incorporating the individual, social (parents and peers), and psychological influences may be the most effective way of reducing hazardous adolescent use of alcohol and other substances [38–41]. In line with this, we will examine the effect of interventions targeting the school, parent, and student level on hazardous use of alcohol (primary outcome) and related health behaviors such as other substance use and well-being (secondary outcomes).

#### School level

In Denmark, the legal age for purchasing products with < 16.5% alcohol is 16 years and 18 years for alcohol with ≥ 16.5% alcohol. Nevertheless, most secondary education programs have social events characterized by adolescents’ heavy and hazardous drinking (alcohol is sold at events arranged by the school; students are typically between 15 and 18 years). Restricting the availability of alcohol has been shown to decrease alcohol consumption in the general population and among college students [39, 42, 43]. Thus, in recent years there has been an increased recognition of how local policies or code of conduct related to alcohol use can create a clear framework for when and how alcohol is or is not part of social events at the school [44]. Some high schools in Denmark have thus imposed local restrictions and policies, for example: alcohol sold at social events at the high school can maximum contain 5% pure alcohol, alcohol is not sold to students younger than 16 years, free bottled water is available, and restrictions are communicated to parents, teachers, and students [38]. The evidence for such approaches is, however, relatively weak, in part because of difficulties related to implementation and evaluation [45]. For example, experiences with university alcohol policies have shown that their implementation can fail if policies are not consistently enforced [46]. Also, school-level policies that are introduced in an environment with high availability of alcohol may have little likelihood of affecting students’ intake [47]. Thus, while the strategies initiated so far on some high schools seem promising, the effect is unknown, and systematic scientific examinations of school alcohol policies’ effects on hazardous use of alcohol in Danish adolescents are warranted.

#### Parent level

Although adolescence is a period characterized by a desire to establish individuality and although adolescents are increasingly oriented towards their peers, parents are not yet out of the picture. The vast majority of adolescents in high school are still living with their families, and parents play an important role as a protective factor e.g., by impacting decision-making around substance use [48]. A wide range of parenting variables, such as parent monitoring, involvement, support, provision of alcohol, quality of communication and parents’ alcohol specific attitudes, and rule setting, have been found to impact adolescent alcohol use behavior [49–51]. Relatedly, reviews and meta-analyses have shown links between adolescent alcohol use and the degree to which parents hold favorable attitudes towards alcohol use, and whether they set alcohol-specific rules, defined as clear instructions or agreements between parents and their adolescent [52, 53]. Further, it has been found that involving parents in alcohol use prevention interventions effectively reduces and prevents adolescent alcohol use [54–57], even if the involvement is of low intensity [58] and consists of information-based approaches centered around single events like parent meetings [59–61]. Interventions targeting parents are absent in Danish high schools, and we lack knowledge on the impact of such interventions on hazardous alcohol use among Danish adolescents.

#### Student level

One of the largest obstacles for individual level prevention programs in Denmark is that the vast majority of adolescents (and their parents [11]) do not perceive heavy adolescent drinking as harmful [12]. Hence, raising awareness, and at the same time offering interventions that do not require youth to see drinking as harmful, are central aspects when developing effective prevention programs, and interventions.

Motivational interviewing (MI) [62] is a directive, strength-based, affirming, non-judgmental, and empathic communication strategy, aimed at resolving ambivalence around a target behavior to foster and support the individual’s intrinsic motivation for behavior change (such as reducing hazardous drinking). Even when utilized in 1–2 sessions, studies from the U.S. show that MI conversations has some of the strongest outcomes for adolescent alcohol and other substance use [40, 63], and for adolescent alcohol prevention specifically [41, 64, 65]. Qualitative evaluations from the U.S. indicate that the MI approach resonates well with adolescents [66].

Our recent pilot study showed that MI administered in groups (group MI) also is highly acceptable with Danish adolescents in high school [67]. The majority of students (82%) reported enjoying the intervention, 80% would recommend group MI to a fellow student, and several began to reflect on their drinking. Furthermore, we found that students in group MI reported significantly greater reductions in peak drinks per drinking day compared to control students, with the largest effect size found for students who reported enjoying the group MI [67].

In general, group-based interventions have been found to have high salience with this age group, likely because of the developmental relevance of including peer community within the intervention [68–70]. Although the effect of *individual* MI on adolescent alcohol and other substance use has received most attention and empirical support [e.g. 71], recent studies reflect that *group* MI also has promising effects on adolescent alcohol and other substance use [64, 69, 72–74].

Thus, grounded in the high prevalence of hazardous drinking and use of other substances in high schools in Denmark, we initiate the large randomized controlled multisite trial ‘Our Choice’ to examine the efficacy of interventions targeting the school, parent, and student level.

### Aim and hypotheses

The main aim of the ‘Our Choice’ randomized controlled multisite trial is to test the efficacy of interventions aimed at reducing hazardous use of alcohol among first year students in high school (ages 15–18). In particular, we will investigate and compare the outcomes of the following conditions:

1. A structural condition with interventions targeting the school and parent level (**structural only condition**).
2. A structural condition combined with a group MI intervention which also targets the student level via group MI (**structural + group MI condition)**.
3. An assessment only control condition with the same assessments as the other two conditions, and with interventions offered after the last follow-up survey (**control condition**).

Furthermore, we aim to examine **feasibility and acceptability** of the interventions with Danish students in high school, as well as with staff (e.g., teachers and principals), to inform future implementation studies.

We will examine the following main hypotheses:

- The **structural only condition** will be superior to the **control condition**. More specifically, we hypothesize that adolescents in the **structural only condition** will have a lower level of hazardous alcohol use (primary outcome), alcohol-related consequences and use of other substances, and a higher level of well-being (secondary outcomes) compared to adolescents in the **control condition** 12 months post baseline.
- The **structural + group MI condition** will be superior to the **structural only condition**. More specifically, we hypothesize that adolescents in the **structural + group MI condition** will have a lower level of hazardous alcohol use (primary outcome), alcohol-related consequences and use of other substances, and a higher level of well-being (secondary outcomes) compared to adolescents in the **structural only condition** 12 months post baseline.
- The **structural + group MI condition** will be superior to the **control condition**. More specifically, we hypothesize that adolescents in the **structural + group MI condition** will have a lower level of hazardous alcohol use (primary outcome), alcohol-related consequences and use of other substances, and a higher level of well-being (secondary outcomes) compared to adolescents in the **control condition** 12 months post baseline.

## Methods

### Trial design

**Fig. 1:**
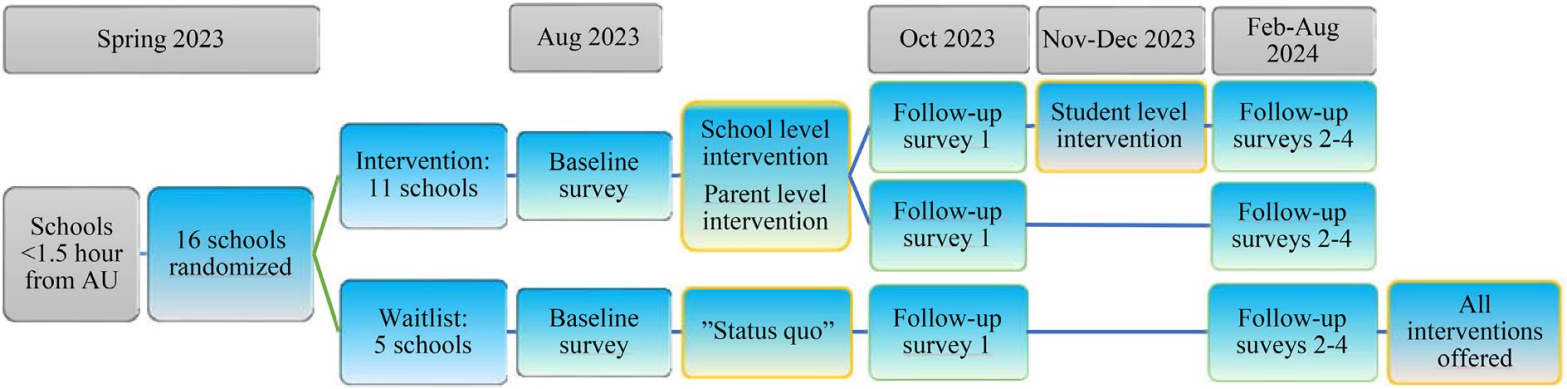
**Study Design with randomization to the three conditions in two steps** In the first step (Spring 2023), 16 high schools were recruited and randomized to a control (5 control high schools) or intervention (11 intervention high schools) condition. In the next step (November 2023), randomization occurs at class-level within the intervention schools, i.e., half of the classes at the 11 intervention schools are randomized to the structural only condition and the other half are randomized to the structural + group MI condition

The trial design is a parallel group cluster randomized controlled trial (cRCT) with three conditions (see Fig. 1):

1. Structural condition with interventions targeting the school and parent level (**structural only condition**).
2. Structural condition combined with group MI condition which also targets the student level (**structural + group MI condition**).
3. Assessment only control condition which includes the same assessments as the other two conditions; interventions are offered after the last follow-up survey (**control condition**).

Furthermore, we use a participatory research design, applying stakeholder theory, which suggests that engaging stakeholders in intervention planning, implementation, and evaluation stages enhances program outcomes [75]. An impactful avenue for improving the development and implementation of interventions is to mobilize both explicit and tacit knowledge from multiple stakeholders and enable collaborative knowledge generation [76]. Shared learning between academic and community knowledge is fundamental for participatory research, which necessitates the establishment of equitable research partnerships with a diverse group of stakeholders – here within school settings, namely school principals, executive assistants, teachers, and students (target group), who impact both the implementation of the interventions and the quality of the research [77–79]. To ensure a high degree of shared learning in all stages of the process from exploring key concepts to designing, testing, and evaluating the interventions, the following are integrated into the trial:

- Prior to the recruitment of schools, members of the target group (high school students) participated in adapting a U.S. group MI intervention to Danish context and setting [67] (the student level intervention in the trial). Via focus groups, students piloted versions of two group MI sessions developed for Danish high school students. This provided students with a relevant and fun way to explore key features of group MI and give valuable feedback on e.g., format, content, and language, which helped ensure that the Danish group MI fit contextually and culturally to a Danish high school setting.
- In collaboration with stakeholders at participating schools (principals, students etc.) policies for school based social events has been developed using a co-creation inspired approach focusing on empowering the stakeholders and giving them opportunities to influence the final intervention (the school level intervention in the trial). A 1-day workshop for representatives of the 11 intervention schools was held Spring 2023. Three researchers, 16 principals and head of studies, and 12 student representatives participated. The aim of the workshop was to facilitate a collaborative process to exchange experience and debate potential elements to include in the shared list of policy-initiatives related to alcohol and social events at the schools, which the schools agreed to enforce in the school year August 2023 to August 2024. This stage in the co-creation process helps ensure that all solutions emerging from the collaborative knowledge generation at the workshop are supported and endorsed by all stakeholder groups.
- Throughout the trial, the research team meets twice yearly with an external expert advisory board consisting of national and local community high school and health experts to help ensure the best conditions for successful study completion and potential future implementation in other school settings. Wider involvement of multiple stakeholder groups including experts on school operations, municipal policies and adolescent health have been identified as important avenues to enhance intervention outcomes and implementation fidelity [78, 80]. Regular feedback from the external expert advisory board will ensure that future versions or elements of the interventions will be contextually appropriate and aligned to school community needs.
- Throughout the trial, members of the research team meet with a youth expert panel consisting of high school students (from schools not participating in the trial). The youth panel participate as ‘expert informants’ by providing perspectives that qualify decisions related to materials used in the trial (e.g., written information about the study, consent forms, materials used in group MI), ensuring that all materials are contemporary, comprehensible and resonates with the student target group. In line with the definitions from The National Council for Children (Danish: “Børnerådet”) [81] the research group will ensure that the youth panel are sincerely listened to, supported in expressing their ideas, that their views are taken into account and inform them about how their involvement will be utilized [82].

### Study setting and recruitment

Publicly funded high schools were contacted by e-mail and invited to hear more about potential participation in the trial until we reached 16 eligible high schools (Spring 2023). The 16 high schools are situated in 13 Danish cities of varying population size: <25.000 (5 schools), 25.000-100-000 (5 schools), 100.000-200.000 (4 schools) and > 200.000 (2 schools). Students starting in August 2023 are invited to take part. Students and their parents/guardians receive participant information minimum one week before enrollment (distributed via the school).

### Eligibility criteria

Publicly funded high schools in Denmark (in Danish: Almen studentereksamen, STX) are eligible if they: (1) are available during the school year August 2023 to August 2024; (2) agree to not initiate other interventions or initiatives related to substance use prevention during August 2023 to August 2024; (3) do not have comprehensive alcohol prevention interventions in place, and (4) are located within 1.5 h drive from Aarhus University.

### Inclusion criteria

A. Students: starting high school in one of the 16 participating publicly funded high schools in August 2023, minimum 15 years old, understand and speak Danish, and able to give independent informed consent.
B. Staff: participating school principals, teachers, or administrative employees, minimum 18 years old, understand and speak Danish, and able to give independent informed consent.

### Conditions

#### Control condition

The control condition is an assessment only condition. During the period under study, it includes the same assessments (surveys) as the other two conditions. After the last follow-up (August 2024), control schools can opt in to receive any of the intervention programs.

#### Structural only condition

The structural only condition consists of: (1) school policies for school-based social events; and (2) an information-based interactive parent meeting.

#### School policies for school-based social events

Schools randomized to the intervention group have agreed on a list of school policies for school-based events aimed at reducing hazardous drinking at, or related to, social events at school premises. The policy-initiatives include (but are not limited to): increasing students’ social cohesion by increasing the number of social events at the school without alcohol (e.g., communal eating, games and theme events); promoting non-alcoholic beverages at parties; working with student associations regarding their roles and responsibilities as role models; encouraging parental involvement at school events (e.g., by inviting parents to be cloakroom attendants at school-parties), and disseminating the schools’ alcohol policy among students and parents. The school policies for school-based social events were developed at a 1-day workshop facilitated by members of the research team in Spring 2023 for representatives of the 11 intervention schools (three researchers, 16 principals and head of studies, and 12 student representatives participated). The schools agreed to enforce the shared list of policy-initiatives related to alcohol and social events at the schools in the school year August 2023 to August 2024. The school principal and management will be responsible for the implementation and enforcement of the policies.

#### Parent meeting

The information-based interactive parent meeting will be 45 min and will take place in August-September 2023, i.e., shortly after the start of the 1st semester. Parents from one/a couple of classes (depending on the size of the school and feasibility) will be invited to attend the meeting. The meeting is physical (in person) and takes place the same day as the schools’ own information meetings for parents. It will entail a 15-minute presentation emphasizing the important role of parents in supporting their adolescents and helping them navigate the challenges related to this developmental period and to starting high school. Parents will be offered concrete, research informed advice about how they can reduce risk of hazardous substance use and promote well-being. To address that culturally, many Danish parents do not see adolescent high intensity drinking as harmful, parents are also informed about practical, real-world frequently reported consequences such as intimate contact or sex that is later regretted; arguments or fights; and increased risk of use of nicotine products and illegal substances. Following the presentation, the group leader facilitates a discussion and exchange of experience between parents based on prepared questions (approximately 30 min). After the meeting, parents will be given a summary of the information to take home (in leaflets).

#### Structural + group MI condition

The structural + group MI condition consists of the elements described above and group MI. Within school classes randomized to receive group MI, participants will be divided into groups with 6–9 participants in each group (depending on class size). The size of the groups is in line with previous clinical trials of group MI by this team [74, 83, 84] including our recent pilot study [67]. Delivering the intervention to all students universally regardless of youth’s individual alcohol use, has several benefits, including maximal reach for the range of adolescent drinking and avoiding inadvertent stigmatizing of adolescents already engaged in drinking.

In Danish high schools, students start in an introductory class in August and change to a permanent class at the beginning of November in the first school year based on choice of study program. Group MI will be delivered in November-December 2023, i.e., after the students start in their permanent class.

The intervention is manualized [85] and consists of two one-hour group sessions administered over two consecutive weeks. The first session is focused on “Tell your story”, “Gains from not drinking”, and “Social norms.” In the second session, focus is on “Personal values”, “Linking values with behavior” and “Planning/choices regarding alcohol”.

Following our recent pilot study [67], the group MI intervention sessions will take place in a classroom at the target schools during the school day. Group MI sessions will be delivered by study staff, trained in group MI. All sessions will be audio-recorded via a digital recorder to ensure MI fidelity and to avoid therapist drift.

### Sample size

Sample size planning was done using simulation in Stata version 17 [86]. Specifically, four level nested data were simulated – levels were repeated measurements (level 1) nested within students (level 2) nested within school classes (level 3) nested within school (level 4). The simulation uniform randomly varied (1) school size between 2 and 11 school classes, and (2) school class size between 25 and 28 students. Random effects for student, school class, and school as well as residual for repeated measurements were randomly sampled from normal distributions with mean zero and variances specified at different values. 33% of sampled schools were assigned to the control condition, whereas school classes in the remaining 66% of sampled schools were assigned 50/50 to either structural only intervention or structural + group MI intervention. The above combined with á priori specified trajectories of alcohol consumption across the four measurements in the three conditions (control, structural only, structural + group MI) were finally aggregated to form the outcome analyzed for power estimation. The simulated outcome was truncated at zero, i.e., if specific simulated outcome was below zero (i.e., indicating “negative number of consumed alcohol units”), the simulated outcome was set to zero.

Finally, to estimate power the above simulated outcomes were analyzed using four-level mixed linear models with random intercept and with interaction between experimental condition and measurement timepoint to allow for different developmental trajectories across the three conditions. Parameters were estimated using restricted maximum likelihood, and for each estimated model, three parameters were tested for significance at alpha = 0.05. The three parameters tested were: 12 months follow-up difference between (1) control and structural only, (2) control and structural + group MI, and (3) structural only and structural + group MI. For a specific set of simulation parameters (i.e., number of sampled schools, variance of random effects and residual, and trajectories of alcohol consumption in each of the three conditions) simulation based power for testing each of the three parameters were estimated as proportion of 1000 estimated models where the specific parameter was statistically significant at alpha = 0.05.

Sufficient power of > 0.80 was reached when including 15 schools assuming 12 months follow-up difference in alcohol consumption (primary outcome) between control condition and structural only condition was 1.5, and between control condition and structural + group MI condition was 3.5, and between structural only condition and structural + group MI condition was 2.0 (further assuming variance of school level = 2, variance at school class level = 2, variance at individual level = 4, and variance of residual = 7).

### Assignment of interventions

KBW generated the allocation sequences via computer generated random numbers in Stata version 17 [86]. First, allocation sequence at school level was randomly generated using uniformly random block sizes of 3 or 6 schools, with 33% of schools allocated to control condition and 66% of schools allocated to intervention condition (Spring 2023). Second, within each school allocated to intervention condition, 50% of school classes will be randomly allocated to the structural only condition and 50% will be randomly allocated to the structural + group MI condition. If a school has an uneven number of school classes, random allocation to intervention-type will be made as close to 50/50 as possible.

The allocation sequence for school level randomization was send to third party not otherwise involved in the study, who, upon request from LVH, would disclose the next number on the allocation sequence to LVH when she was about to inform a particular school what condition it was allocated. Since block sizes were random, it was not possible for LVH to predict the next allocation number.

Shortly after students have changed to their permanent class (start of November 2023), a school specific school class allocation list as described above will be generated for each school allocated to the intervention condition. LVH will disclose this list to each individual school principal. The random school class allocation to either the structural only condition or the structural + group MI condition will therefore be disclosed simultaneously for all school classes at a particular school. After assignment to interventions, blinding is not possible.

### Assessment of outcomes

Survey data from the students will be collected at baseline, and 2-, 6-, 9-, and 12- months post baseline. The survey-questionnaires will be digital and time for filling out the questionnaires will be allocated during school hours. Members of the research team will introduce the surveys in person and are present in the classroom during data collection to answer any questions that may arise concerning the study, the survey, or the participants’ rights. This may facilitate retention in the trial. Furthermore, the link to the survey will be open the following 1–2 days to allow students absent on the day of testing to complete the survey.

#### Primary and secondary outcomes

The primary evaluation of efficacy is based on outcomes measured 12 months post baseline.

We will examine the effect of the interventions on the following primary outcome:

1. Past month high intensity drinking: peak drinks per drinking event, assessed with the Timeline FollowBack (TLFB) [87].

We will examine the effect of the interventions on the following secondary outcomes:

1. Past month alcohol-related consequences, assessed by 21 items covering physical, social and mental issues related to hazardous use of alcohol, developed by the team for Danish youth based on previous Danish surveys and the Rutgers Alcohol Problems Index [88] e.g. black-outs, (emotional) hangovers, sexual contact that was later regretted, with yes/no response options (summed score).
2. Past month heavy episodic drinking events assessed by TLFB (count measure).
3. Well-being in class assessed by six items rated on a five point scale from ‘completely disagree’ to ‘completely agree’ e.g., *I feel accepted by the others in my class* (inspired by [89]) (summed score).
4. Psychological well-being, assessed by The World Health Organization Five Well-being Index (WHO-5) [90] (summed score).
5. Past month use of nicotine products (days using nicotine products; count measure), and past month use of illegal substances (days using cannabis, cocaine, and other illegal drugs; count measure).

### Assessment of implementation fidelity, feasibility, and acceptability

Assessment of implementation fidelity, feasibility, and acceptability will be done in the following ways:

#### School policies for school-based social events

In order to measure the degree to which the school policies for school-based social events have been implemented, all students (from intervention and control schools) will be asked to assess to which degree they experience that the different policies apply to their school. Hence, as part of the surveys students will be asked “how often do you find that the following applies at your high school” followed by e.g., (1) students below 16 can buy alcohol at the school’s parties, or (2) invitations to the schools parties encourage heavy drinking. This will be assessed at baseline, 2-, 6-, 9-, and 12 months post baseline.

#### Parent meeting

To evaluate feasibility of the interactive parent meeting, we will evaluate attendance rates. To evaluate acceptability of the parent meeting, we will examine parent satisfaction with the meeting, including whether they found the information relevant, and whether they would recommend the school to offer this meeting to parents next year (i.e., parents of first year students). These measures will be administered at the end of the parent meeting via an anonymous link.

#### Group MI

To evaluate feasibility of the group MI, we will evaluate enrollment. To evaluate acceptability of the group MI sessions, we will examine whether the students liked taking part and whether they would recommend it to other students. Furthermore, we will use open-ended questions to examine what they liked, what could be improved and what made an impression on them [67]. Acceptability measures of group MI will be administered to students in the structural + group MI condition shortly after the last group MI session.

In line with our recent pilot study of group MI for adolescents [67], researchers delivering group MI will be trained in the MI manual and practice (by Kristine Rømer Thomsen and an experienced external MI trainer). All sessions will be audio recorded for MI integrity and fidelity evaluation; (1) for supervision during the trial to prevent therapist drift, and (2) for post-hoc evaluation of MI integrity and fidelity by the MI Coding Lab at University of Southern Denmark. A total of 30% of all conducted sessions will be assessed, using an established metric of MI fidelity [91], similar to the procedure in our pilot trial. The fidelity of MI will be measured by means of Motivational Interviewing Treatment Integrity manual version 4.2.1 (MITI 4) [91]. MITI 4 measures 10 different behavioral counts and four global scores. The behavior counts are: *giving information, question, simple reflection, complex reflection, affirmation, seeking collaboration, emphasize autonomy, persuade with permission, persuade*, and *confront*. The four global scores are: *cultivating change talk, softening sustain talk, partnership*, and *empathy* [91]. The constructs in the MITI 4 have demonstrated acceptable interrater reliability [92].

Furthermore, we will investigate the implementation fidelity of the school, parent and student level interventions using qualitative interviews with a random sample of staff involved in the project at intervention schools (principal and relevant teachers/other staff). Interviews will be done after the last follow-up survey and will be focused on the perception of the interventions, how participation in the interventions was perceived and suggestions for improvement of the interventions.

### Data management

Quantitative data from the surveys will be entered into a SurveyXact server hosted on a secured server in Denmark. Quantitative data from the randomization, enrollment, and the surveys, audio recordings of the group MI sessions as well as qualitative data from the interviews will be kept on a secure server hosted by Aarhus University. At the end of the trial, the quantitative data will be uploaded to a server on Statistics Denmark if we deem it appropriate to merge data with national registers. The MI Coding Lab at University of Southern Denmark will code 30% of all conducted group MI sessions via a secure website: https://app-kodningslab.cloud.sdu.dk/. All audio recordings of group MI sessions will be deleted after coding and analysis. For all other data, only current and future members of the research team, who have a research affiliation to Aarhus University, will have access to the data.

### Statistical analyses

All descriptive analyses, the consent rate, the participation rate at the interactive parent meetings, the participant rate at each of the two group MI sessions, the completion rate of all surveys, and potential study drop-out will be reported and summarized after the quantitative data collection has ended. All of this information will be provided in a CONSORT flowchart.

Summaries will be presented as means and standard deviation of variables that are approximately normally distributed, or as medians and IQRs for skewed variables. Categorical variables will be summarized as frequencies and percentages.

The main objective of the statistical analyses is to assess: (a) the superiority of the structural only condition versus the control condition on hazardous alcohol use 12-months post baseline; (b) the superiority of the structural + group MI condition versus the structural only condition on hazardous alcohol use 12-months post baseline; and (c) the superiority of the structural + group MI condition versus the control condition on hazardous alcohol use 12-months post baseline.

As the primary outcome is a count measure of alcohol use, where we expect extra zeros due to students not drinking for e.g., age- or religious reasons, we plan to analyze the main objectives ideally using zero inflated generalized linear mixed models with poison family and log link-function. In case the zero inflated generalized linear mixed model cannot converge, we will instead evaluate the main objectives using a zero inflated (normal) linear mixed model. In either case, random effect will be added for intercept (i.e., clustering of longitudinal measurements within students), whereas higher order clustering will be accounted for using robust standard errors.

Secondary outcomes will be analyzed using mixed models to account for the nested data-structure. Depending on the distribution of the specific outcome either (normal) linear mixed models or generalized linear mixed models with poison family and log link-function, and potentially zero-inflated. We expect well-being measures to be somewhat normally distributed, whereas count of alcohol related consequences, count of heavy drinking episodes, and count of days using tobacco or illegal substances will somewhat follow a zero-inflated poisson distribution.

Analyses of efficacy (primary and secondary outcomes) will be based on the intention-to-treat sample, utilizing all available follow-up data from all randomized participants. All randomized participants will be analyzed within the condition they were allocated after randomization, regardless of whether they complete the intervention or not. Participants who withdraw their consent for use of their data during the trial period will not be included in any of the analyses.

No interim analyses and stopping guidelines will be applied, because the prevention interventions of the trial do not entail risk of harm to the participants.

Results from the trial will be published in international peer-reviewed journals, preferably with open access, and in national outlets with open access. Furthermore, presentations of main findings are offered to participating schools and other interested stakeholders.

## Discussion

The overall aim of the ‘Our Choice’ trial is to test the efficacy of interventions aimed at reducing hazardous use of alcohol among first year students in Danish high schools (ages 15–18). By conducting a fully-powered cluster-randomized multi-site controlled trial with three conditions we will be able to examine the effects of interventions targeting the school and parent level (structural only condition) compared to an assessment only condition and examine potential additional effects of also targeting the student level (structural + group MI condition) – on hazardous use of alcohol (primary outcome) and related health outcomes (secondary outcomes) in high school students.

To our knowledge, the trial is the first (internationally) to compare the efficacy of a structural intervention targeting the school and parent level to an intervention targeting these levels and the student level via group MI – on hazardous drinking and related health outcomes among students.

The potential effectiveness of these interventions holds significant implications for the health and well-being of adolescents, influencing multiple facets of their lives. Addressing hazardous alcohol use during adolescence is of paramount importance due to growing evidence showing that heavy drinking during adolescence negatively impacts cognition and brain structure [93], and is associated with alcohol-related accidents and negative experiences (e.g., sex which is later regretted) [4], and with increased risk of a range of adverse outcomes such as use of illegal substances [37], juvenile delinquency [94], poorer academic achievements [95], and alcohol-related health risks in adulthood [96, 97].

Implementing effective, feasible and acceptable prevention interventions in salient settings – such as high schools – are of great importance. If effective, the tested interventions (school policies for school based social events, parent meeting, and group MI) can be implemented at low cost. The school policies for school based social events are of almost no cost to the schools, and personnel at schools can be training in delivering the parent intervention at very low cost. Similarly, group MI can be implemented at low cost by training personnel from the schools (e.g., student counsellors) to deliver it. The research team members delivering group MI in the pilot trial [67] and the current randomized controlled trial receive minimal training (3 day course with external MI supervisor and internal training in the manual). The group MI training has been kept to a minimal to ensure future implementation.

In conclusion, the trial ‘Our Choice’ offers valuable novel insights on the efficacy of prevention interventions targeting the school, parent and student levels on hazardous drinking and related health outcomes among high school students. The study has significant implications for adolescent health and potential to impact evidence-based decisions on alcohol prevention policy, education, and health professions.

### Electronic supplementary material

Below is the link to the electronic supplementary material.

Supplementary Material 1

Supplementary Material 2

## Data Availability

After the conclusion of the study, data from the study will be made available by the Centre for Alcohol and Drug Research, Aarhus University, upon reasonable request, and within the limitations set by the Danish data protection legislation and regulation.

## Declarations

### Contact for public and scientific queries

Kristine Rømer Thomsen, Aarhus University, Denmark, e-mail: krt.crf@psy.au.dk.

### Public title

Our choice.

### Data monitoring

A data monitoring committee is not needed because the prevention interventions included in this trial do not entail risk of harm to the participants.

## List of abbreviations

Group MI: Group based motivational interviewing
