# Supplementary material for: Prevention of hazardous use of alcohol among high school students: a study protocol for the randomized controlled trial ‘Our choice’
Source: BMC Public Health. 2023 Oct 24;23:2079. doi: 10.1186/s12889-023-16976-y (PMC10594784; doi:10.1186/s12889-023-16976-y)
Supplement: Supplementary file 1 — Supplementary Material 1 [file 12889_2023_16976_MOESM1_ESM.docx]

# Invitation to participate in the research project: our choice

­

**Dear student (and parents)**

This academic year, [high school name] has agreed to participate in an exciting research project for 1st-year high school students – **Our** **Choice**. Aarhus University is conducting the project in close collaboration with your school and other high schools. We hope you will support this project and that you are interested in participating.

You are receiving this information because, as part of the project, we must inform participants about the purpose of the project and what participation entails.

The project is based on previous national and international studies. The purpose of the project is to investigate how student communities, well-being and substance use are connected. It also explores how we can best support good social communities and promote well-being and health (e.g., regarding the use of substances like alcohol), and how the physical and social contexts around Danish high school students may play a role in this.

**What is going to happen?**

You are invited to participate in the academic year 2023-2024. Your participation will take place during school hours, ensuring that you will not have any absences and you won’t need to spend extra time on it after school hours. What your participation entails is as follows:

- You are invited to complete online questionnaires five times during the next 12 months (from August 2023 to August 2024). The questionnaires include questions about your well-being, health and potential use of alcohol and other substances. You will fill out these questionnaires during school hours.

Participating is voluntary, so it’s up to you whether you want to be involved. Participation only occurs after you (and your parents) have received information about the project and have agreed to participate. If you do not wish to participate, there will be no consequences. Everyone who is invited to participate in a research project has the right to consider it beforehand. Therefore, we kindly ask you and your parents to read this material and discuss it thoroughly before you make your decision.

If at any point you no longer wish to participate, you can discontinue your involvement in the project and the questionnaire surveys.

**Data processing**

The project is approved by the Research Ethics Committee at Aarhus University (ID BSS-2023-039) and by the Danish Data Protection Agency. The processing of your data will be handled in accordance with current legislation. This means that your answers are **confidential,** and the information you provide will only be used for research and dissemination of research results. Your information will always be presented in aggregated summaries and results ­­­­– for example, in a graphical representation of the results. This means that it is impossible to identify you, or your school, based on this information.

We encourage you (and your parents) to read more about how Aarhus University handles your data in the document "How We Process Your Personal Information" below.

All data are stored in a secure system at Aarhus University, accessible only to researchers working on the project. The information is stored for as long as it is relevant for the research. There may be follow-up research or other projects where your data could be used. When it's no longer relevant to have data that can be traced back to you, the data will be anonymized.

Results from the project will be published in scientific journals and will be continuously made easily accessible at www.crf.au.dk. It will not be possible to identify you, your school, or other participants in the publications.

**Ethical considerations**

There are no known risks associated with participating in the project. If, however, you experience any problems or discomfort as a result of your participation, please let us know, so we can address it.

The project started in February 2023 and will be running for four years. The project is supported by TrygFonden with 7 million DKK and is led by Associate Professor and psychologist Kristine Rømer Thomsen from the Department of Psychology, Aarhus University.

We look forward to your participation in the project, and you are always welcome to contact us if you need more information.

Kind regards,

Synnøve Xylander, Lotte Vallentin-Holbech and Kristine Rømer Thomsen

Contact information:

Project employee, Ph.d.-student, Synnøve Xylander, e-mail: [sx.crf@psy.au.dk](mailto:sx.crf@psy.au.dk)

Project coordinator, Assistant Professor, Lotte Vallentin-Holbech, e-mail: [lvh.crf@psy.au.dk](mailto:lvh.crf@psy.au.dk)

Project leader, Associate Professor, Kristine Rømer Thomsen, e-mail: [krt.crf@psy.au.dk](mailto:krt.crf@psy.au.dk)

Centre for Alcohol and Drug Research, Institute of Psychology

Aarhus BSS, Aarhus University
Bartholins Allé 10, building 1322
8000 Aarhus

# Invitation to participate in the research project: our choice

­

**Dear student (and parents)**

This academic year, [high school name] has agreed to participate in an exciting research project for 1st-year high school students – **Our** **Choice**. Aarhus University is conducting the project in close collaboration with your school and other high schools. We hope you will support this project and that you are interested in participating.

You are receiving this information because, as part of the project, we must inform participants about the purpose of the project and what participation entails.

The project is based on previous national and international studies. The purpose of the project is to investigate how student communities, well-being and substance use are connected. It also explores how we can best support good social communities and promote well-being and health (e.g., regarding the use of substances like alcohol), and how the physical and social contexts around Danish high school students may play a role in this.

**What is going to happen?**

You are invited to participate in the academic year 2023-2024. Your participation will take place during school hours, ensuring that you will not have any absences and you won’t need to spend extra time on it after school hours. What your participation entails is as follows:

- You are invited to complete online questionnaires five times during the next 12 months (from August 2023 to August 2024). The questionnaires include questions about your well-being, health and potential use of alcohol and other substances. You will fill out these questionnaires during school hours.
- Some classes will be invited to participate in group discussions in November or December 2023 (You will receive more information if this applies to you).
- Your parents will be invited to participate in a dialogue meeting with other parents from your class. The meeting will focus on how they can create good conditions for young people’s well-being, health, and positive communities.

Participating is voluntary, so it’s up to you whether you want to be involved. Participation only occurs after you (and your parents) have received information about the project and have agreed to participate. If you do not wish to participate, there will be no consequences. Everyone who is invited to participate in a research project has the right to consider it beforehand. Therefore, we kindly ask you and your parents to read this material and discuss it thoroughly before you make your decision.

If at any point you no longer wish to participate, you can discontinue your involvement in the project and the questionnaire surveys.

**Data processing**

The project is approved by the Research Ethics Committee at Aarhus University (ID BSS-2023-039) and by the Danish Data Protection Agency. The processing of your data will be handled in accordance with current legislation. This means that your answers are **confidential,** and the information you provide will only be used for research and dissemination of research results. Your information will always be presented in aggregated summaries and results ­­­­– for example, in a graphical representation of the results. This means that it is impossible to identify you, or your school, based on this information.

We encourage you (and your parents) to read more about how Aarhus University handles your data in the document "How We Process Your Personal Information" below.

All data are stored in a secure system at Aarhus University, accessible only to researchers working on the project. The information is stored for as long as it is relevant for the research. . There may be follow-up research or other projects where your data could be used. When it's no longer relevant to have data that can be traced back to you, the data will be anonymized.

Results from the project will be published in scientific journals and will be continuously made easily accessible at www.crf.au.dk. It will not be possible to identify you, your school, or other participants in the publications.

**Ethical considerations**

There are no known risks associated with participating in the project. If, however, you experience any problems or discomfort as a result of your participation, please let us know, so we can address it.

The project started in February 2023 and will be running for four years. The project is supported by TrygFonden with 7 million DKK and is led by Associate Professor and psychologist Kristine Rømer Thomsen from the Department of Psychology, Aarhus University.

We look forward to your participation in the project, and you are always welcome to contact us if you need more information.

Kind regards,

Synnøve Xylander, Lotte Vallentin-Holbech and Kristine Rømer Thomsen

Contact information:

Project employee, Ph.d.-student, Synnøve Xylander, e-mail: [sx.crf@psy.au.dk](mailto:sx.crf@psy.au.dk)

Project coordinator, Assistant Professor, Lotte Vallentin-Holbech, e-mail: [lvh.crf@psy.au.dk](mailto:lvh.crf@psy.au.dk)

Project leader, Associate Professor, Kristine Rømer Thomsen, e-mail: [krt.crf@psy.au.dk](mailto:krt.crf@psy.au.dk)

Centre for Alcohol and Drug Research, Institute of Psychology

Aarhus BSS, Aarhus University
Bartholins Allé 10, building 1322
8000 Aarhus C

Consent Statement

**Title of the Research Project: our choice**

Identification Number, Research Ethics Committee, Aarhus University: (ID BSS-2023-039)

Participant consent statement:

I have received comprehensive information about the project, and I have sufficient knowledge about the purpose, method, benefits, and drawbacks to give my consent to participate.

My participation in the project involves answering five electronic questionnaires over the next twelve months.

I am aware that participation is voluntary, and I can always discontinue my participation in the questionnaire surveys without losing any of my current or future rights.

I consent to participate in the research project and have received a copy of this consent form, as well as a copy of the written information about the project for my personal use.

Participant name: _____________________________________________________________________

Date: __________________ Signature: ___________________________________________________

Consent Statement

**Title of the Research Project: our choice**

Identification Number, Research Ethics Committee, Aarhus University: (ID BSS-2023-039)

Participant consent statement:

I have received comprehensive information about the project, and I have sufficient knowledge about the purpose, method, benefits, and drawbacks to give my consent to participate.

My participation in the project involves answering five electronic questionnaires over the next twelve months and participating in two group discussions lasting approximately one hour each, with other students from my class.

I am aware that participation is voluntary, and I can always discontinue my participation in the group discussions and questionnaire surveys without losing any of my current or future rights.

I consent to participate in the research project and have received a copy of this consent form, as well as a copy of the written information about the project for my personal use.

Participant name: _____________________________________________________________________

Date: __________________ Signature: ___________________________________________________
